# A Case of Paroxysmal Hæmaturia, and Effects of Physostigma in Convulsive Diseases of Children

**Published:** 1877-04

**Authors:** 


We find on our table two short papers from the pen of Dr. G. S. Trezevent; one on "a case of paroxysmal hæmaturia," and the other on "effects of physostigma in convulsive disease of children."

In the first-named monogram we are reminded of the various phases assumed by malarial disease. Hæmaturia and hæmatomesis seem to comprise the forms of hemorrhagic difficulty attending the forms of miasmatic fever. Malignancy, the result of a more virulent quality of the poison, leads to that more devitalized condition of the tissues, allowing the slow escape of blood through the mucous membranes. Hence, black vomit and hæmaturia are found in yellow fever and the hæmaturial fever of Southwestern Georgia and Southern Alabama.

Dr. Trezevent found quinine the great spinal tonic, to relieve the "paroxysmal hæmaturia," of which his paper treats, and takes the proper view of the pathological condition of the spinal and sympathetic system of nerves in the production of all forms of malarial disease.

In the paper on the "effects of physostigma," we have confirmation of the benefit derived from calabar bean in the treatment of reflex convulsive disease.

